# Supplementary material for: Perspectives of adolescents and young people on Digital Health Interventions and their impact on health knowledge
Source: PLOS Glob Public Health. 2026 Apr 7;6(4):e0005611. doi: 10.1371/journal.pgph.0005611 (PMC13056157; doi:10.1371/journal.pgph.0005611)
Supplement: S1 Appendix — (DOCX) [file pgph.0005611.s001.docx]

**S1. Focus Group Discussion Recruitment Script**

**A User-centered Approach to Designing a Social Media App Fostering Interaction, Peer Learning and HIV Awareness Among Adolescents and Young People in a Resource-Limited Setting; Kibra & Kikuyu, Kenya.**

**Adolescent Recruitment Script**

My name is ______________; I am a researcher from Kenyatta National Hospital. We are interested in finding out what information adolescents need to help them with the following issues: HIV prevention, mental health, disclosure, substance use, stigma, reproductive health and intimate partner violence from a sample of adolescents in your locality. By doing so, the study will know your information needs and gaps. We will also request you to suggest how to use of a number of buttons/features of a social media app so that they meet your needs. We value what you are going to say. Please remember that your participation is voluntary and you are free to not respond to any question or to end the interview at any time.

The interview will last approximately 1 hour. You will receive **Kshs. 500** for your time and effort if you agree to be part of the interview.

We are asking for your permission to record the interview to be transcribed and analyzed afterwards. The recording will be locked up away in a safe place and the final reports will not be traceable back to individual participants.

Would you be interested in participating in a discussion with other adolescents to provide your experiences and perspectives? **Yes/No**

Are you 19 - 24 years old? **Yes/No**

Are you a resident in this community? **Yes/No**

If **YES TO ALL ABOVE**: Proceed to the consent process.

If **NO TO ANY**: Thank you for your time [END ENROLLMENT].
