# Supplementary material for: Perspectives of adolescents and young people on Digital Health Interventions and their impact on health knowledge
Source: PLOS Glob Public Health. 2026 Apr 7;6(4):e0005611. doi: 10.1371/journal.pgph.0005611 (PMC13056157; doi:10.1371/journal.pgph.0005611)
Supplement: S2 Appendix — (DOCX) [file pgph.0005611.s002.docx]

## **S2. Focus Group Discussion Guide**

**Baseline Survey**

**A User-centered Approach to Designing a Social Media App Fostering Interaction, Peer Learning and HIV Awareness Among Adolescents and Young People in a Resource-Limited Setting; Kibra & Kikuyu, Kenya.**

**Focus Group Discussion Guide**

**Facilitator’s welcome, introduction, and instructions to participants**

My name is ______________; I am a researcher from Kenyatta National Hospital. As mentioned, our discussion and co-design activities will take about 1 hour. Please, give your honest responses to the questions. We know you could be busy, so we will make the discussion as productive as possible. Do you have any questions before we start? [ADDRESS ANY QUESTIONS]

**Opening questions**

1. In your view, how many hours do adolescents like you use the internet weekly?
   1. Why this number of hours?
   2. What are the adolescents accessing on the internet?
2. Do you think that you and your peers use social media to get information about your health?
   1. If so, how have adolescents used social media apps to get information about their health?
   2. If they don’t use, are there benefits of adolescents using social media apps to get information about their health?

**Topic 1: Health information on social media**

1. Did you trust the information about your health that you accessed on social media apps?
   1. Why or why not?
2. How do adolescents like you access a social media app providing health information?

**Topic 2: Discussion point on social media app**

1. What would you recommend to be discussed in the social media app on:
   1. Mental health? Why?
   2. HIV? Why?
   3. Sexual reproductive health? Why?
   4. Substance use? Why?
   5. Intimate partner violence? Why?
   6. Nutrition? Why?

**Topic 3: User roles, confidentiality and ground rules**

1. Which persons (adolescents, healthcare providers, researchers, policy makers) would you be comfortable to interact with in the social media app? Why?
2. What role should healthcare providers have in the social media app?
3. How could your privacy and confidentiality be protected in the social media app?
4. Which ground rules would you recommend in the social media app forum?

**Topic 4: Co-design activities**

**Session two, in groups of 3 adolescents**. (*Facilitator, divide the FGD participants into groups of 3 participants. Provide pencils and writing materials*)

Now we are going to discuss each of the following social media app feature. Feel free to suggest how these features could be used to make the social media app useful to you.

Sketch envisioned interaction by the users for each feature.

**Chat**

The feature provides a chat/instant messaging feature. Start chatting from the profile icon of community site member, or by selecting a friend from the friend-picker on the chat toolbar. Sessions will be shown on the chat toolbar.

**Features:**

- Privately chat with other members in the community,
- User option: only your friends, everyone or nobody is able to invite you to chat,
- Multiple members / friends in one chat session,
- Multiple sessions at the same time,

**Site announcements**

Tell your site users about something important.

**Questions**

This is a feature that allows users to ask questions.

**Features**

- Ask questions as an individual or to a group
- Comment on questions + answers
- Like questions + answers
- Assign experts to help answer questions

**Poll**

A Poll is a feature that can be created on a timeline or story. This feature allows you to ask a question and give answers in a multiple-choice format, to choose from, for users. Users can click on the answer they want, from the choices you created or add their own answer if you give them that option.

A deadline on the poll, after which users can not submit their answers anymore. After the poll is over, the results are shown against each answer – how many votes did an answer get and what percentage does it constitute.

**Blog**

A blog is the internet’s way of indulging with curious readers. It’s a place where individuals (or groups of them) may voice their thoughts and share them with other users.

**Debates**

This plugin provides a place where anyone can open threads on any subject, creating separate spaces where people can discuss the proposed topic. Debates are valued by everybody, to highlight the most important issues.

The debates module makes it easier for users to meet each other to debate or collaborate on these issues. Users can vote for or against the debates, so that the most highly valued debates are those that are regularly displayed on the main debates page.

**Features**

- Create debates
- Users can vote for or against a debate
- Comments section where users can debate with each other
- Highly valued debates will be available in the home page

Thank you for your time. Now you will proceed to the registration desk to receive your reimbursement.
