# Supplementary material for: Perspectives of adolescents and young people on Digital Health Interventions and their impact on health knowledge
Source: PLOS Glob Public Health. 2026 Apr 7;6(4):e0005611. doi: 10.1371/journal.pgph.0005611 (PMC13056157; doi:10.1371/journal.pgph.0005611)
Supplement: S3 Appendix — (DOCX) [file pgph.0005611.s003.docx]

**S3. Informed Consent Form Focus Group Discussion**

**Baseline Survey**

**A User-centered Approach to Designing a Social Media App Fostering Interaction, Peer Learning and HIV Awareness Among Adolescents and Young People in a Resource-Limited Setting; Kibra & Kikuyu, Kenya.**

**Adolescent Informed Consent Form**

| **Name** | **Institution** | **Qualification** | **Contact** |
| --- | --- | --- | --- |
| Paul Macharia | Kenyatta National Hospital | Bsc, Msc, PhD | +254722866753 |
| James Serembe | University of Nairobi | Bsc, MA(Ongoing) | +254722267588 |
| Cyrus Mugo | Kenyatta National Hospital | MBChB, MPH, PhD | [cyrusmugodr@gmail.com](mailto:cyrusmugodr@gmail.com) |
| Christine Ngaruiya | Yale University | MD, MSc, DTM&H | [christine.ngaruiya@yale.edu](mailto:christine.ngaruiya@yale.edu) |
| Violet Wanjihia | Kenya Medical Research Institute | PhD | [vwanjihia@gmail.com](mailto:vwanjihia@gmail.com) |
| David Bukusi | Kenyatta National Hospital | MBChB, MMed | [davidbukusi@gmail.com](mailto:davidbukusi@gmail.com) |
| Lawrence Ikamari | University of Nairobi | PhD | [likamari@uonbi.ac.ke](mailto:likamari@uonbi.ac.ke) |
| Ruth Nduati | University of Nairobi | MBChB, MPH | [ruth_nduati2000@yahoo.com](mailto:ruth_nduati2000@yahoo.com) |

**Introduction**

Hello, my name is --------------------. **You** have been invited to take part in a research study. Before you decide whether to **participate** or not, you need to understand why the research is being done and what it would involve. Please take the time to read or to listen as I read the following information. You may talk to others about the study if you wish. Please ask me if there is anything that is not clear, or if you would like more information. When all of your questions have been answered and you feel that you understand this study, you will be asked if you **will participate** in the study, and if you decide to **Participate** in this research study, we will then ask you to give your **written consent**. Your **participation** is completely voluntary and **you** can withdraw participation at any time with no penalty.

**Purpose of the Study and Study Requirements**

**What is the study about?** This study is being carried out by **Paul Macharia from Kenyatta National Hospital**, the study seeks to identify information adolescent like **you** and your needs on HIV prevention, mental health, disclosure, substance use, stigma, reproductive health and intimate partner violence from a group of adolescents in your locality. By doing so, the study will be able know your information needs and gaps and how best to provide the information using a web-based social media app. The reason we are carrying out this study is to improve the health and well-being of adolescents like ***you.***

**Why have you been invited to take part?** You have been invited to take part because **you** are an adolescent living in this neighborhood where the study is taking place.

**What will happen if I take part?**

1. **You** will be enrolled into a group discussion with other adolescents to identify adolescent questions or information on HIV prevention, mental health, disclosure, substance use, stigma, reproductive health and violence.

**Risks**

**What are the risks of participating in the study?** You may feel embarrassed or uneasy taking part in discussions on HIV prevention, mental health, disclosure, substance use, stigma, reproductive health and violence.

**Benefits**

**What are the benefits of participating in the study?**

You will have an opportunity to be provided with more information on HIV and the services available here, your participation will provide information on how to design a web-based social media app aimed to enable adolescents connect with each other, share what they are going or have gone through and learn from other adolescents on what works for them on HIV prevention, care, treatment and support which could improve experience when you visit a health facility.

**Confidentiality**

**Will participation in the study be kept confidential?** We will take all steps necessary to ensure **what you say** will not be known by others outside the study. Given that **you** will be visiting study site venues for registration, **your** attendance may be known to other people who are also participating in this study. However, we will request all participants to keep information that they observe about others during the visits confidential and not to share it with anyone.

**You** will be given a unique number and **your** name will not appear on any information like answers to the questions we ask you on the phone. We will do our best to protect you and confidentiality of all data will be strictly enforced. All data will be kept in a secure location and will be destroyed at the end of the study. Each participant will respect the participation of others at the study site venue.

**Voluntariness**

**What are my rights as a research participant/subject?** **Your** participation in this study is completely voluntary. It’s not a **must** for you to take part. Even after you decide **to take part**, **you** may end **your** participation at any time without being punished or loss of existing services to which **you are** entitled.

**Involuntary Early Termination of Participation:**

Participants may be stopped or discontinued from the study if those providing money to do the research, government or the organization ensuring we are doing the research in the correct way may stop the study before its planned end date.

**Additional Information**

**What will I receive for participating? You** will not be paid for participation in the study, but you will receive **Kshs. 500** as transport reimbursement and your time answering the questions, we asked you.

**Will it cost me anything to participate?** No, you will not be required to pay anything to be in the study.

**What will happen to the results of the research study?** The results of the study will be discussed and presented in a report at team meetings and it will be used to help the design of mobile phone apps about our health. The information may be presented at a conference with other studies that relate to social media-based apps about our health. Neither the report nor the presentation will show **your** name. The study findings will also be written to be read by other people who work in adolescent health.

**Who has reviewed the study for ethical issues?** This study has been reviewed and approved by the **Kenyatta Hospital/University of Nairobi Ethics Review Committee**.

**What if I need more information?** If you have questions about any aspect of the study, you can ask me now or you can speak to any of the study staff who will do their best to answer your questions. If you have any other questions about the study, you may also **call Paul Macharia on +254-722-866753** or **email** [**paulmachariah@gmail.com**](mailto:paulmachariah@gmail.com)

**What if there is a problem?** Any complaint about the way **you** have been treated during the study or any possible harm you might suffer will be addressed. **Please contact the secretary of The Kenyatta National Hospital/University of Nairobi Ethics Review committee on +254-020-2726300.**

At this time, I want to make sure you understand the study and see whether you have any additional questions. After we discuss any questions you may have, we can discuss whether you are interested in participating in the study today.

**Are you willing to participate in the study?**

No ☐ **(if no, stop here)**

Yes ☐ **(if yes, continue below)**

**Study staff who conducted informed consent discussion:** I confirm that I have personally explained the nature and extent of the planned research, study procedures, potential risks and benefits, and confidentiality of personal information.

**Participant’s name (print(Upper case)) Participant’s**

**signature/thumbprint and date**

| **Study staff conducting consent discussion (print)** |  | **Study staff signature and date** |
| --- | --- | --- |

| **Witness’s name**  **appropriate)** | **(print)** | **(if** |  | **Witness’s appropriate)** | **signature** | **(if** |
| --- | --- | --- | --- | --- | --- | --- |
