# Supplementary material for: Perspectives of adolescents and young people on Digital Health Interventions and their impact on health knowledge
Source: PLOS Glob Public Health. 2026 Apr 7;6(4):e0005611. doi: 10.1371/journal.pgph.0005611 (PMC13056157; doi:10.1371/journal.pgph.0005611)
Supplement: S4 Appendix — (DOCX) [file pgph.0005611.s004.docx]

**S4. Coding Tree for the Deskes qualitative analysis studying health issues among AYPs in Kenya and their recommendations for a health app**

| **Themes** | **Subthemes** | **Quotes** |
| --- | --- | --- |
| HIV | Prevention | R3: “For me I would prefer if they discuss about the family planning they should also be able to provide PrEP, PEP all those because you can find you are talking about it and you do not know how it looks like so before we talk about those issues we should know the samples”  R6: “...nowadays there is that self-test kit, for me I feel that they are supposed… what is supposed to be talked about is how to use that kit. Most of us we don't know, personally I have never used, I don't know how it operates…”(FGD5) |
|  | Transmission | R3: “....If someone has had sex with a person who has HIV then immediately go and have sex with someone else can the second person get HIV?Like the way a man goes to the lodging and has sex with a woman then the same day goes and has sex with his wife…” (FGD4) |
|  | Stigma | R3: “I think what many youths are lacking, or what we are lacking, is the information, more information about HIV. We've grown up saying, oh, this person has HIV, don't even talk to them, but you see that stigma. We've grown up to places that you see a person with HIV, you see a person is slim you think they have HIV, you've grown in a place like, if you see a person being positive and you sit there then you will be positive” (FGD6) |
|  | Side Effects | “Talk of the side effects that HIV can bring to you personally in terms of health even if you use those drugs also how can it affect other people around you, even your families and other people close to you maybe(FGD7) |
|  | Living with HIV | “...if I'm already HIV positive, if I'm already pregnant, what is the journey from there on? How will you help me?..(FGD6) |
|  | Living with HIV infected people | “How can we live positively with these people because some people have the mind that they can’t share even a meal with these people if they know them so if today I know somebody who has this disease I can try as much as possible to unfriend such a person so how can we avoid this? We have the mentality that I can contract HIV anytime.” (R3, FGD7) |
| Sexual Health | Menstruation | “They should teach our females in Kibra how to keep hygiene like when you walk in Kibera you will see pads disposed everywhere.” (R2, FGD11) |
|  | Teen Mother/Teen Pregnancy | “In early pregnancy you find that she does not even have the skills she is still a child she does not know what she needs to do at each stage, at times you may find that she is supposed to give birth on that day but she does not know she is just in the house she does not have the knowledge.” (R4, FGD11) |
|  | STI’s | “We are aware that these STIs are not only brought by sex, it also depends on cleanliness. So, the adolescents need to be aware on how to do the cleaning.” (R6, FGD1) |
| Nutrition |  | “I think it is relevant for you to have information about nutrition firstly most of the people don’t know the importance of healthy nutrition habits or the dietary requirements in that line and we end up having poor eating habits, for example I will eat chapati on my way then when I get home I take rice and that is more starch and maybe in the day I have taken starch so it might be really of help in that line and also it may provide the quantity needed and all that because you may eat a whole pack of food but it does not meet the nutritional requirements for your health” (R3, FGD8) |
| Violence | Interpersonal violence | “What I can say about that is, for violence it cannot get to a point you butcher someone like that at one day, it starts with slapping someone once and it escalates from there so for us as women and men we are both precious, someone should lay his hands on you and you keep quiet about it, you should report that person or look for a way to resolve it and if you stay with someone who is just hitting you and sometimes it is not only hitting you sometimes it is through word of mouth, someone is verbally abusing you and you feel that he is abusing you even by bringing your family as well, that is not good so if you see someone mistreating you, you need to report” (R4 FGD9) |
| Substance use |  | “R5: I would suggest having information on how to get rehabilitation centers that are close to you to reduce the risks that the drug user may bring to other people. So, to have accessibility of rehabilitation centers.” (R5 FGD1) |
| Mental health | Stress | “On mental health I think one of the things that should be discussed is stress and depression. I think it is one of the things that is really affecting young people nowadays because; the generation we are in doesn't like speaking out what is going on in their lives. So what they think is there like is disturbing their minds they can't say it, like others can't tell friends. ” (R4, FGD5) |
|  | Death / suicide | “Mental health is an issue because nowadays you hear people can kill a person something I find to be so difficult so I can think this person is not well upstairs so it is an issue so we need ways....” (R4, FGD7) |
| Ground rules of app | Age limit | “R: …It would be best if there was every age group  M: What do you mean every age group?  R: Even the big people they have their issues” (R6, FGD10) |
|  | Etiquette | “People should not write anything that is offensive or will hurt someone else” (R4, FGD1) |
|  | Privacy | “You make sure the information that you put there is confidential.” (R2, FGD4) |
| Use of social media |  | “Adolescents like me don’t get information on health on the internet because they want things that are entertaining or things that will give them followers, maybe on Facebook, those fun things.” (R3, FGD3) |
| Reasons to search internet/use social media apps for health information |  | “Sometimes in the social media you can find someone has said about a problem that he has and is looking for comments from others.” (R1, FGD7) |
| Other reasons for using internet/social media | Watching videos  Searching for jobs | …you can get a challenge like lack of money, credit you get a challenge of bundles to use internet (R1, FGD7) |
| Existing health-specific apps |  | “I think let’s say most of the youths especially those who are a bit educated they visit online platforms like Google to get information about health, maybe something is happening to you and maybe you don’t want to visit a health care practitioner so it is cheap and convenient to ask Google you get the health information you require even before visiting a health practitioner.” (R4, FGD8) |
| Cost of app | Charges for access of social media apps  Other accessibility issues | “you can get a challenge like lack of money, credit you get a challenge of bundles to use internet.” (R1, FGD7) |
| Features | Presentation of Information  Advertisements on app  Tailoring | “the app should be should be like personal oriented, like it should not be generalized in terms of…..it should treat anyone using the app as an individual” (R5, FGD5) |
| Trusted sources of health information | Peers  Experts  Role on app | “Sometimes you don’t use apps on social media, you go straight to Google, you Google and then it gives you an answer.” (R4, FGD9) |
