# Supplementary material for: Perspectives of adolescents and young people on Digital Health Interventions and their impact on health knowledge
Source: PLOS Glob Public Health. 2026 Apr 7;6(4):e0005611. doi: 10.1371/journal.pgph.0005611 (PMC13056157; doi:10.1371/journal.pgph.0005611)
Supplement: S5 Appendix — (DOCX) [file pgph.0005611.s005.docx]

**S5. Recommended features of a future health app for youth in Nairobi Kenya**

| **Recommended Features** | **Quotes** |
| --- | --- |
| Free access | “M: Okay something else…let's say you ask something and you have not been answered well yet I think I have answered you would you want a contact where you can ask this and should it be free or you pay?  Chrus: Free  R4: If you ask us to pay we will not use <<<laughter>>>  M: So you are saying at least for the period we are testing the App it should be free and to call whoever expert you want to call is also free?  Chorus: Yes”(R4, FGD12) |
| 24/7 availability | “When would you like it to be open to you?  All: 24-7”  (All, FGD7) |
| An alarm for people living with HIV to remind them to take their medications | “On that, I would also like them to add the app to have some kind of an alarm that if I am positive I will be able to set my time. I take my medicine at six in the morning so I'll be able to… I'll be able to set six in the morning, it reminds me to take my medicine.” (R2, FGD5) |
| Multimedia content | “I think, because it's for young people, maybe you can use…because it's for young people, maybe you can use a lot of photos, videos or even podcasts. Because a young person just reading about mental health, depression is the state of... It's so tiresome and cumbersome. In fact, most of them will just open the first line and then switch off, so they make it interesting for them. Maybe, I think podcasts will really do, because right now very many young people are in podcasts.” (R2, FGD6)  “R: I will say that when it is written every time the youth neglect it, if it was information about HIV then I get there it is written 10 pdfs I will skip it so short videos or short voice notes concerning a certain topic like HIV that will entice the youths to access the Apps more.  M: Not PDFs very good…  R: If they are PDFs they be short ones.” (R2, FGD7) |
| Referral system | “For the App I would like to have an App where I can book an appointment with the doctor where so that I can also be confident enough about confidentiality, privacy you notice in a hospital set up and you don’t know who you are going to meet maybe parent lecturer and you want to check your status maybe HIV somebody sees you entering the VCT maybe it is your relative <<<laughter>>> at the end of the day you may end up ruling off going to that place whereby you can book an appointment interact with a doctor and all that.” (R6, FGD8) |
| Telemedicine consultation/counseling capability | “I will also suggest that the app is more on mental health focus on specific individualized one person such that if a person maybe wants to open up about a certain issue you can do a video call with admin or health care worker or you can do a phone call where you can do a one on one because you know some of these services people pay for them, so the youths want something convenient something you can do when you are free and you just want to open up about something and you just want to talk to somebody who does not know you but open in such a way that you feel relieved.” (R4, FGD8) |
| Map/directions to nearby healthcare facilities | “On the App I can add that we can have a map that will direct somebody to the facility that they may be needing that is near them, maybe if I traveled from here to some other parts then I need a facility, maybe rehabilitation or pharmacy then I don’t know where to start looking for it so if we can have a map.” (R3, FGD8) |
| Reminder capability | “If there can be a way that it can have a reminder, let's say right now, I am busy, but I want to find certain information in the evening. Maybe I can set a reminder on the App.” (R5, FGD8) |
| Ability to order from a nearby pharmacist | “I want to add that the app should be organized in such a way that maybe a youth can be able to order something let’s say… maybe somebody needs a condom and you are fearful in approaching the health center to pick a condom or go and buy in a chemist you can order like give me a box of condoms and they are delivered <<<laughter>>> these things they are there, or going to do a HIV test you know going to the chemist and saying you want to do a HIV test that person will look at you like this, so if the App can provide youth centered products that they can order and they will be delivered then it will be more beneficial to the youths in terms of health.” (R5, FGD8) |
| Accountability tracking system like receipts for services provided | “Also, to add on that if there can be provision of receipt or something maybe I have engaged the doctor and gotten a certain service and I can be able to get a receipt maybe I made a purchase or something for recording purposes and also accountability.” (R6, FGD8) |
| Photo/video upload capability | “R2: Then there is an important feature we forgot to put on the app you should ensure there is somewhere you can upload a photo like you have diarrhea and you want to show the doctor you take a picture  R?: You vomited  R2: Yeah, the kind of vomit you have  R?: I am coughing, rashes  R2: Uploading pictures and videos is very important” (R2, FGD8) |
