# Supplementary material for: Perspectives of adolescents and young people on Digital Health Interventions and their impact on health knowledge
Source: PLOS Glob Public Health. 2026 Apr 7;6(4):e0005611. doi: 10.1371/journal.pgph.0005611 (PMC13056157; doi:10.1371/journal.pgph.0005611)
Supplement: Attachment — Submitted filename: Reviewer_Response_Letter_PLOS_Global_Public_Health_20251228.docx [file pgph.0005611.s006.docx]

Maiya G Block Ngaybe
1914 ½ E 1st St
Tucson, AZ 85719
mgblock@arizona.edu
Phone: +1-520-841-0829

December 28, 2025

Editorial Team
PLOS Global Public Health

Editors and reviewers,

We are writing to thank you and the reviewers for the additional feedback regarding our manuscript titled *“Perspectives of adolescents and young people on Digital Health Interventions and their impact on health knowledge.”*

Below, we have provided a table that summarizes each of the reviewers' comments along with our corresponding responses and the changes made to the manuscript.

We once again appreciate the opportunity to improve our work. We hope that the revised manuscript meets the standards of PLOS Global Public Health and look forward to your feedback.

Please do not hesitate to contact us if any further information or clarification is needed. We thank you for considering our submission and are grateful for the time and effort you have invested in our manuscript.

Sincerely,

Maiya G Block Ngaybe

| **Comment** | **Response** |
| --- | --- |
| **Journal Requirements** | |
| 1. Please include a complete copy of PLOS’ questionnaire on inclusivity in global research in your revised manuscript and upload a completed version as Supporting Information. | We completed PLOS’ questionnaire on inclusivity in global research (included below), and uploaded the completed questionnaire as Supporting Information. |
| 2. Please amend your detailed Financial Disclosure statement so that it is in full sentences, includes author initials alongside each funding source, and states what role the funders took in the study. | We revised the Financial Disclosure statement to be written in full sentences, specify the initials of the author(s) associated with each funding source, and state the role of the funders in the study. (Lines 879-883) |
| 3. Please ensure that your Ethics Statement is available in its entirety at the beginning of your Methods section, under a subheading 'Ethics Statement'. | We moved the complete Ethics Statement to the beginning of the Methods section under the subheading “Ethics Statement.” (163-167) |
| 4. Please upload separate figure files in .tif or .eps format, and remove the figures from your manuscript file but keep the legends. | We removed the figures from the manuscript file while retaining the figure legends, and uploaded each figure as a separate .tif file in accordance with the PLOS guidelines. (591-594) |
| 5. Please remove Appendix 1, 2, 3, 4 from the manuscript file and upload them as Supporting Information, and list each Supporting Information file after the references. | We removed Appendices from the manuscript file and uploaded each as separate Supporting Information files. |
| 6. Please add a full list of legends for your Supporting Information files after the references list. | We added a complete Supporting Information legends section after the references list. (1032-1039) |
| 7. Data availability: All PLOS journals require all data underlying the findings to be freely available unless public deposition would breach ethics board protocol. Please provide an explanation and request an exemption if needed. | We revised the Data Availability statement to align with PLOS policy. Because public deposition of full qualitative transcripts could compromise participant privacy and conflict with the approved protocol and consent language, we request an exemption for public release of verbatim transcripts. To maximize transparency, we provide the coding framework/codebook and additional de-identified supporting excerpts as Supporting Information, and we will share further de-identified data with qualified researchers through an ethics-consistent controlled access process upon request. (869-877) |
| **Reviewer 1** | |
| Abstract: Methods What type of qualitative study was this (Narrative, phenomenological or …)? | We clarified the study design in the Abstract and Methods as a qualitative descriptive study using focus group discussions and thematic analysis. (23-25, 183) |
| Can you justify use of focus group discussion to study the sensitive topic among adolescent and young people? | We added a brief justification for use of focus group discussions and described safeguards used for sensitive topics (e.g., trained facilitators, confidentiality ground rules, private venues, and participant language choice). (175-176, 223-225) |
| Abstract: Results Clarify the themes that you found in your study. I find number of ideas explained not clear if these are the found themes or otherwise. | We revised the Abstract Results to clearly summarize the major themes identified in the analysis. (32-37) |
| Introduction You have done great job reviewing literature from globally to East Africa on second paragraph. What about data from study country (Kenya)? | We strengthened the Introduction by adding Kenya-specific context and citations relevant to adolescents and young people’s SRH information needs and the local HIV/SRH landscape. (112-116) |
| Refer to original document with the respective information for example reference 1, refer to “UNAIDS. The Path That Ends AIDS. 2023 UNAIDS Global AIDS Update. Available at: https://thepath.unaids.org/  wpcontent/themes/unaids2023  /assets/files/2023_report.pdf. [Accessed 27 April 2024]” instead of “Rakhmanina N, Foster C, Agwu A. Adolescent and young adults with HIV and unsuppressed viral load: where do we go from here?” | We updated the reference list and in-text citations to cite the original UNAIDS 2023 Global AIDS Update document where appropriate. (898-900) |
| Methods Study area What is age range for adolescents? Then this statement “The study team worked with community-based organizations in these areas to recruit adolescents aged 19 – 24 years.” | We clarified age definitions and eligibility throughout the Methods. We define adolescents as 10–19 years and young people as 19–24 years, and we corrected the wording to reflect that the study enrolled participants aged 19–24 years (young people). (182-189) |
| Methods Data collection At what time was English and Swahili used? Since you used both languages | We clarified in the Methods that discussions were conducted in English and/or Swahili based on participant preference. (237-239) |
| Why was data saturation not discussed? | We added a brief description of the saturation approach and clarified that data collection continued until thematic saturation was reached across sites. ( |
| Results The authors should revise this sentence for clarity. It is not clear whether females were predominantly unemployed, and males were predominantly students, or if another interpretation is intended. Additionally, several characteristics (education, employment, internet access) are combined in one long sentence. I recommend restructuring into multiple clearer statements. | We revised the Results demographic description for clarity by splitting the long sentence into multiple statements and aligning it with Table 1. (331-337) |
| In table 1 row 2, it states Age (Median), what is the median age and the number in brackets (19-23)? What is the unit that measured age? Is it years or months? One suggestion: Age (Years), Median (IQR) | We revised Table 1 to specify age in years and to present age as “Age (years), median (IQR)” and clarified the values accordingly. (Table 1) |
| Discussion Revise the discussion to include all the themes, compare, contrast the findings, and include possible explanations. | We revised the Discussion to explicitly address all key themes, compare and contrast findings with prior literature, and include plausible explanations and implications for intervention design and HIV/SRH programming in Kenya. (750-798) |
| **Reviewer 2** | |
| 1. Adolescent Involvement in the Participatory Approach While the paper notes that a participatory approach was applied, it only briefly mentions the level of adolescent involvement beyond their participation as study subjects. I recommend adding a more detailed description of how adolescents were engaged throughout the research process. This could include clarification on whether they were involved in co-designing data collection tools etc. | We expanded the Methods to more clearly describe how adolescents and young people were engaged through a participatory approach, including how their input shaped discussion activities and intervention feature recommendations. (192-230) |
| 2. Gender-Specific Discussion The study commendably highlights differences in perspectives between male and female participants; however, this component is not sufficiently addressed in the discussion section. Given well-documented gender differences in health-seeking behaviors and digital engagement patterns, it would strengthen the paper to discuss how these findings might inform the design of gender-responsive features within the proposed digital health intervention (DHI). | We strengthened the Discussion to more directly interpret gender differences and translate these findings into gender-responsive DHI design implications (e.g., privacy/confidentiality features, trusted sources, and tailored content and engagement strategies). (771-798) |

**Supporting Information Legends**

S1 Text. Recruitment script used to introduce the study and screen participants.

S2 Text. Focus group discussion guide (Session 1 and Session 2 co-design activities).

S3 Text. Participant information sheet and informed consent form.

S4 Text. Coding tree / codebook used for thematic analysis.

S5 Text. Recommended features of a future digital health app for youth in Nairobi, Kenya.

S6 Checklist. PLOS Inclusivity in Global Research Questionnaire (completed).

Inclusivity in Global Research Questionnaire (Completed)

**Ethical considerations, permits and authorship**

Q1. Provide details as to who granted permissions and/or consent for the study (e.g., ethics review board approval, local administrative approvals) and describe how participant consent was obtained.

A1. Ethical approval was obtained from the Kenyatta National Hospital/University of Nairobi Ethics Review Committee (P655/09/2023). Recruitment was conducted with youth-serving community-based organizations and in private spaces within church compounds hosting adolescent-friendly services. All participants provided written informed consent prior to participation, in English or Swahili.

Q2. If there were any deviations from the study protocol after approval was granted, please describe them.

A2. No protocol deviations relevant to participant protections or data collection procedures were made after ethics approval (or, if minor operational changes occurred, these are described in the Methods).

Q3. Did this study involve local collaborators who are residents of the study country or community? If yes, briefly describe their roles.

A3. Yes. The study was led and implemented by investigators based in Kenya (University of Nairobi and Kenyatta National Hospital) and conducted in collaboration with youth-serving community-based organizations in the study sites, who supported recruitment and advised on locally appropriate engagement.

Q4. Please confirm that all listed authors meet the journal’s authorship criteria and describe any relevant contributions or collaborations.

A4. All listed authors meet PLOS authorship criteria and contributed to study conception, design, data collection and/or analysis, interpretation, and manuscript drafting and revision.

Human subjects research

Q5. Did you obtain written informed consent from a representative of any underage participants? If not applicable, mark N/A.

A5. N/A. This qualitative study enrolled young people aged 19–24 years.

Q6. How did members of the local community provide input on the investigation, its methodology, and its anticipated outcomes?

A6. Participants contributed through participatory FGDs that functioned as co-design sessions, identifying priority health information needs and recommending content and features for a youth-centered digital health intervention. Community-based partners provided input on recruitment and culturally appropriate study procedures.

Q7. When engaging with the local community, how did you ensure that the study’s aims, procedures, and materials could be understood by local stakeholders?

A7. Study materials were written in plain language and delivered in English and/or Swahili. Facilitators were bilingual and reviewed confidentiality and group ground rules at the start of each discussion.

Q8. Will the findings be made available in an understandable format to local communities and stakeholders? Please provide details.

A8. Yes. The study team will share plain-language summaries with participating community-based organizations and adolescent-friendly service providers in the study sites and will present findings to local stakeholders through dissemination meetings and/or stakeholder workshops.

Non-human subjects research (specimens/animals/materials): N/A.
